# Synergistic effects of a cremophor EL drug delivery system and its U0126 cargo in an *ex vivo* model

**DOI:** 10.1080/10717544.2019.1636421

**Published:** 2019-07-05

**Authors:** S. T. Christensen, A. S. Grell, S. E. Johansson, C. M. Andersson, L. Edvinsson, K. A. Haanes

**Affiliations:** a Department of Clinical Experimental Research, Copenhagen University Hospital, Rigshospitalet-Glostrup, Copenhagen, Denmark;; b GenuiNova AB, Hjärup, Sweden;; c Department of Clinical Sciences, Division of Experimental Vascular Research, Lund University, Lund, Sweden

**Keywords:** Basilar artery, cremophor EL, endothelin-1, MEK1/2, optimal dose, U0126

## Abstract

Neuroprotection has proven clinically unsuccessful in subarachnoid hemorrhage. We believe that this is because the major component in the early damage pathway, the vascular wall, has not been given the necessary focus. U0126 is a potent inhibitor of vascular phenotypical changes, exemplified by functional endothelin B (ET_B_) receptor upregulation. The current study aimed to determine the optimal dose of U0126 *ex vivo* and test the toxicology of this dose *in vivo*. To find the optimal dose and test a suitable *in vivo* delivery system, we applied an *ex vivo* model of blood flow cessation and investigated functional ET_B_ receptor upregulation (using a specific agonist) as the primary endpoint. The secondary endpoint was depolarization-induced contractility assessed by 60 mM K^+^ stimuli. Furthermore, an *in vivo* toxicology study was performed on the optimal selected doses. U0126 (10 µM) had a strong effect on the prevention of functional ET_B_ receptor contractility, combined with minimal effect on the depolarization-induced contractility. When cremophor EL was chosen for drug delivery, it had an inhibitory and additive effect (combined with U0126) on the ET_B_ receptor contractility. Hence, 10 µM U0126 in 0.5% cremophor EL seems to be a dose that will be close to the maximal inhibition observed *ex vivo* on basilar arteries, without exhibiting side effects in the toxicology studies. U0126 and cremophor EL are well tolerated at doses that have effect on ET_B_ receptor upregulation. Cremophor EL has an additional positive effect, preventing functional ET_B_ receptor upregulation, making it suitable as a drug delivery system.

## Introduction

Stroke is ranked as the second largest cause of death worldwide after coronary heart disease, and it accounts for almost 1.1 million deaths each year in the European Union (Warlow et al., 2003). More than four decades of research on neuroprotection has proven clinically unsuccessful (O'Collins et al., 2006). We believe the reason for this is that the major component involved in the early injuries – the vascular wall – has not been given the necessary focus (Edvinsson & Povlsen, 2011). Our research has so far shown that stroke (focal/global ischemia and subarachnoid hemorrhage) triggers an injury response in the vascular wall of brain arteries/arterioles that evolves over several hours/days, and it has a major impact on the worsening of the initial damage with effects on stroke outcome. We have focused on the initial ‘switch-on mechanism’ after stroke, and our results show that preventing/ceasing the activation of the early vascular injuries is a novel treatment approach (Edvinsson & Povlsen, 2011). The cessation of blood flow in an artery/arteriole is perceived by endothelial cells and subsequently by vascular smooth muscle cells, which promote changes in cellular adhesion molecules of the vessel wall (Spray et al., 2016). This loss of shear stress triggers an intracellular injury cascade which involves the MEK-ERK1/2 signaling pathway, resulting in transcription of some contractile receptors, cytokines and blood-brain barrier (BBB) breakdown proteins (Vikman & Edvinsson, 2006; Henriksson et al., 2007; Maddahi et al., 2011; Grell et al., 2017).

U0126 is an inhibitor of the MAPK/ERK kinase (MEK1/2), which is part of the mitogen-activated protein kinase (MAPK) pathway. U0126 is a specific inhibitor of phosphorylation by MEK1 and MEK2 at IC_50_ 0.07 µM and 0.06 µM, respectively (Duncia et al., 1998; Wityak et al., 2004). The activation of the MAPK pathway in cerebrovascular tissue results in enhanced expression of contractile receptors, such as endothelin type B (ET_B_), 5-hydroxytryptamine type 1B (5-HT_1B_), angiotensin II type 1 (AT_1_), and thromboxane A2 (TX_A2_) in the cerebral arteries (Hansen-Schwartz et al., 2003a,b; Ansar et al., 2007; 2010), as well as in intraparenchymal micro-vessels (Spray et al., 2017). Sarafotoxin (S6c), a highly specific agonist (10,000 fold selective for ET_B_ over ET_A_), has been proven a particularly useful tool in regards to investigating the ET_B_ receptors (Davenport et al., 2016). Timely treatment with U0126 prevents the upregulation of these vasoactive elements, which are believed to have a key function in the debilitating secondary cerebral vasospasm and delayed cerebral ischemia observed after subarachnoid hemorrhage (SAH) (Edvinsson et al., 2014; Macdonald, 2014; Christensen et al., 2019). It has previously been shown with western blot that an increase in contractile responses to the ET_B_ agonist S6c coincided with an increase in ET_B_ protein both *ex vivo* (Li et al., 2012) and in *vivo* (Povlsen et al., 2013).

According to the scientific literature, U0126 is found to be selective for MEK1/2 (Favata et al., 1998; Davies et al., 2000; Bain et al., 2007; Uitdehaag et al., 2012). Its activity is characterized as being noncompetitive with respect to the MEK substrates such as ATP and ERK1/2, and it is also suggested to have a binding preference for the non-phosphorylated form of MEK1/2 (Favata et al. 1998; Davies et al. 2000). Previous studies applying U0126 as an intrathecal treatment has used dimethyl sulfoxide (DMSO) as a solvent for the weakly polar U0126 (Larsen et al., 2011; Maddahi et al., 2011; Povlsen & Edvinsson, 2015). However, DMSO was long ago shown not to be suitable as a vehicle and for drug delivery of human therapies (Smith et al., 1967) and still remains a concern (Weaver et al., 2016). Therefore we looked at other clinical studies involving MEK1/2 inhibitors and found an abundance of available data, in particular from the cancer field, from studies where cremophor EL often is used as a vehicle and for drug delivery (Gelderblom et al., 2001).

The current study aimed to determine the optimal dose of U0126 *ex vivo* and to test the toxicology of this dose *in vivo*. We applied an *ex vivo* organ culture model of blood flow cessation in the rat basilar artery (BA) and investigated functional ET_B_ receptor upregulation as the primary endpoint. The secondary endpoint was depolarizing induced contractility, assessed by a depolarizing 60 mM K^+^ stimuli. We therefore, aimed to determine any potential effects of cremophor EL (also known as Kolliphor EL and from here on termed cremophor) as a drug delivery system of U0126 in the same *ex vivo* model. Finally, we present a toxicology study in rats, which include the dose chosen as the optimal treatment dose, when based on the *ex vivo* data.

## Methods

### Animals for *ex vivo* studies

For the organ culture data, 40 male Sprague-Dawley rats (NTac:SD) purchased from Taconic (Denmark) was used. The animals were maintained at a 12/12-h light-dark cycle (with dark beginning at 7 am) and housed at a constant temperature (22 ± 2 °C) and humidity (55 ± 10%), with food and water *ad libitum*. Rats were generally housed in Eurostandard cages (Type VI with 123-Lid) 2–6 together. All procedures were performed at the Department of Clinical Experimental Research and were approved by the Danish animal ethics committee.

### Artery harvest and organ culture

The rat basilar artery was used to determine the concentration-response relationship *ex vivo* and to determine the lowest effective concentration of U0126. U0126 was dissolved in DMSO to a final concentration of 10 mM. This formulation of U0126 was further diluted with DMEM (Dulbecco’s modified Eagle’s media) to obtain a concentration of 100 µM from which 1, 3, 10 and 30 µM solutions were prepared with paired DMSO controls. In the experiments with cremophor, a stock solution of 1% was prepared in DMEM and further diluted to the desired concentrations. For the U0126 in cremophor, a 200 µM stock in 1% cremophor (sonicated for 45 min to create micelles) was created and further diluted to create the desired concentrations.

BAs were carefully dissected from the brains and cut into 4 equal sized segments (∼1.2 mm long). The segments were incubated for 48 h in DMEM with U0126, DMSO or cremophor, and the media was changed after 24 h. After 48 h of incubation, the segments were used for *ex vivo* pharmacology studies.

### *Ex vivo* pharmacology

For measurements of contractile responses, a wire myograph (Danish Myograph Technology A/S) was used to record the isometric tension in segments of isolated arteries (Mulvany & Halpern, 1977; Hogestatt et al., 1983). Vessel segments were mounted on two 40 μm-diameter stainless steel wires and immersed in a temperature-controlled physiological buffer solution (37 °C) of the following composition (mmol/L): NaCl 119, NaHCO_3_ 15, KCl 4.6, MgCl_2_ 1.2, NaH_2_PO_4_ 1.2, CaCl_2_ 1.5, and glucose 5.5. The buffer was continuously aerated with 5% CO_2_ to maintain a pH of 7.4 and the wires were separated for isometric pretension at 2 Nm^−1^ and were then allowed to equilibrate at this tension for 30–45 min. The segments were exposed to a 60 mM potassium buffer obtained by a partial substitution of NaCl with KCl in the above-described isotonic buffer.

Subsequently, concentration-response curves were obtained by the cumulative application of the specific ligand for the ET_B_ receptor, Sarafotoxin 6c (S6c, Polypeptide, Sweden), in the concentration range of 10^−14^ to 10^−7 ^M (Henriksson et al., 2004).

### Cell counting kit 8

Cell counting kit 8 can be used to measure cellular metabolism (Haanes et al., 2012). The compound in the kit is bioreduced by cellular dehydrogenases to an orange formazan product that is soluble in tissue culture medium. The amount of formazan produced is proportional to the cellular metabolism. Fresh basilar arteries were isolated from the brain, and immediately submerged in 110 µL DMEM media containing 10 µL Cell Counting Kit 8 (DOJINDO) and left in the incubator for 3 h. After 48 h in organ culture, the same basilar arteries were subjected to the same procedure. Absorbance was measured at 450 nm (using 650 nm as a reference) in a micro-plate photometer (Tecan, Infinite M200, software SW Magellan v.6.3, Männedorf, Switzerland).

### Data analysis of *ex vivo* pharmacology

Data are expressed as mean ± standard error of the mean (S.E.M.), and *n* refers to the number of rats in each group. Data were analyzed by comparing 95% confidence intervals for the log EC_50_ values when comparing sensitivity, by multiple unpaired *t*-tests corrected for multiple testing using the Holm-Sidak method (U0126/DMSO) or one-way ANOVA with Holm-Sidak post-test (to control) when comparing concentration effects (U0126/DMSO cremophor). All the analysis was done using GraphPad 8.02 software (San Diego, CA). The relative log EC_50_/log IC_50_ is by four-parameter non-linear regression and is the concentration corresponding to a response midway between the estimates of the lower and upper plateaus. The E_max_ is the maximal contraction in the concentration-response curve. Contractile responses of each segment were adjusted according to the length of the artery and are expressed as mN/mm (Nm^−1^).

### Animals for the toxicology

Seventy-two SD-HLA® (SD)CVF® (Sprague-Dawley equivalent) outbred rats (*Rattus norvegicus*) were purchased from Hilltop Lab Animals, Inc. (Scottdale, PA). Animals were purchased with approximate weight ranges of 230–250 g and 180–200 g for the males and females, respectively on arrival. The animals were obtained with an intracerebroventricular (i.c.v.) cannulae preimplanted by the vendor. Animals were individually-housed due to the i.c.v. cannulae in plastic static micro-isolator cages. General procedures for animal housing and husbandry met all regulations concerning use of animals in research including the U.S. Department of Agriculture regulations (9 CFR Ch. 1) implementing the Animal Welfare Act (7 USC 2131 et seq.) and the recommendations of the National Research Council’s Guide for Care and Use of Laboratory Animals (National Academy Press, 2011). This study was conducted according to a research proposal approved by the Institutional Animal Care and Use Committee of Comparative Biosciences, lnc.

### Toxicology

The study consisted of four groups of 32 rats (16 males and 16 females). Each group was dosed i.c.v. with either 0.5% cremophor alone or test article (U0126 in 0.5% cremophor) at 0.057 µg, 0.57 µg, and 1.14 µg in a dose volume of 15 µL per dose containing 0.5% cremophor. The doses (µg) correspond to 1 µM, 10 µM, and 20 µM in the injected volume, respectively. Animals were dosed twice daily (approximately every 12 h) on day 0–3, and once daily on day 4–7. In-life assessments included a daily clinical observation, body weight measurements (one measurement before the first dose, a weekly measurement and at necropsy), a weekly food consumption, and a functional observation battery test (FOB). Approximately 90 min after the last single dose on day 7, the animals were subjected to terminal blood collection and subsequently euthanized and necropsied. Histological evaluation of hematoxylin and eosin-stained tissue sections was performed on the brain from all the animals and included evaluation of the injection site (the right lateral ventricle), the contralateral (uninjected) left lateral ventricle)) and of four additional brain levels. A full list of organs was also evaluated histologically for all dose groups. Formulations were made fresh every four days and kept refrigerated until use.

## Results

### Optimal concentration of U0126

**Figure 1.**
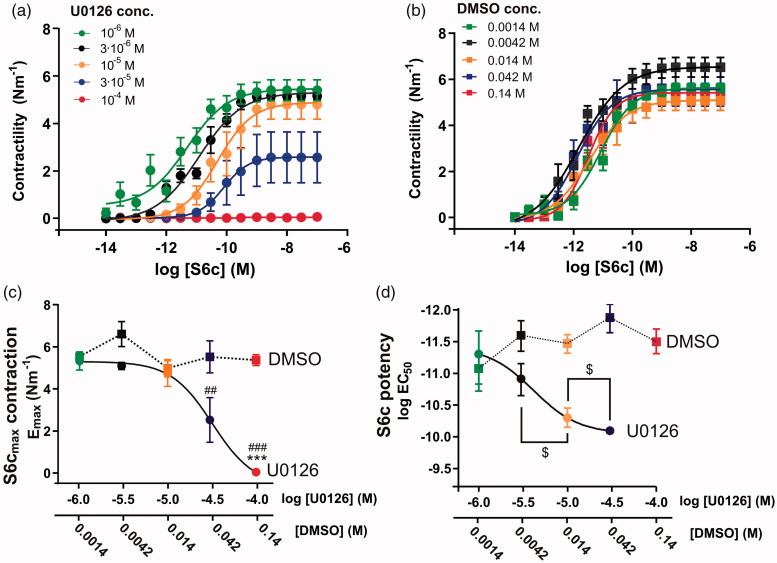
Concentration-dependence of U0126 (and DMSO) on S6c contractility after 48 h organ culture. Basilar arteries were incubated with different concentrations of (a) U0126 and corresponding concentrations of (b) DMSO. (a,b) Concentration-response curves to S6c show varying EC_50_ and E_max_ values. (c,d) Correlation between the concentrations of either U0126 or corresponding concentrations of DMSO (on the double x-axis) of E_max_ (c) or log EC_50_ (d) from the concentration-response curves with S6c (a,b). Values are listed in the Table S1, one-way ANOVA with Holm-Sidak post-test (##*p* < .01 and ###*p* < .001) or multiple *t*-tests with Holm-Sidak correction for multiple testing (***p < .001), $=non-overlapping 95% confidence intervals. Data are expressed as mean ± SEM, *n* = 4–15.

We initially set out to investigate the optimal dose of U0126 on the inhibition of functional ET_B_ receptor upregulation in an organ culture model (Henriksson et al., 2004). We incubated arteries with five different concentrations of U0126 in DMSO (Figure 1(a)) and the paired concentrations of DMSO (Figure 1(b)). We further analyzed the effect of U0126 and DMSO on the E_max_ and log EC_50_ for S6c (Figure 1(c,d)). There was a significant difference of E_max_ between U0126 and DMSO at 10^−4 ^M of U0126 compared to the DMSO control (*p* < .001, Table S1). In addition, 3 × 10^−5 ^M U0126 was significantly lower than 10^−6 ^M U0126 (*p* < .001), but not compared to its paired DMSO control. For the log EC_50_ values for S6c, we did not observe any inhibitory effect of 10^−6 ^M U0126, while at 3 × 10^−6 ^M of U0126 there was a significant shift in the log EC_50_ value and further significant shifts at every half-log increase in the U0126 concentration (no 95% CI overlap, Figure 1(d)). For 10^−4 ^M U0126, the curve was practically flat, making us unable to determine the log EC_50_. Data comparing the U0126 concentration with the corresponding DMSO concentration can be found in the Table S1.

We calculated the pIC_50_ values for U0126 to evaluate the compounds ability to shift the S6c concentration-response curves at the different concentrations. For DMSO we did not observe any concentration dependencies (Figure 1(c,d)), but for U0126 there was a concentration-dependence for the S6c E_max_ contraction (log IC_50_, −4.68 to −4.31 log M, Figure 1(c)), which was almost 10-fold lower than the log IC_50_ for the S6c potency (log IC_50_, −5.55 to −5.21 log M, Figure 1(d)).

### Cremophor drug delivery system in organ culture

**Figure 2.**
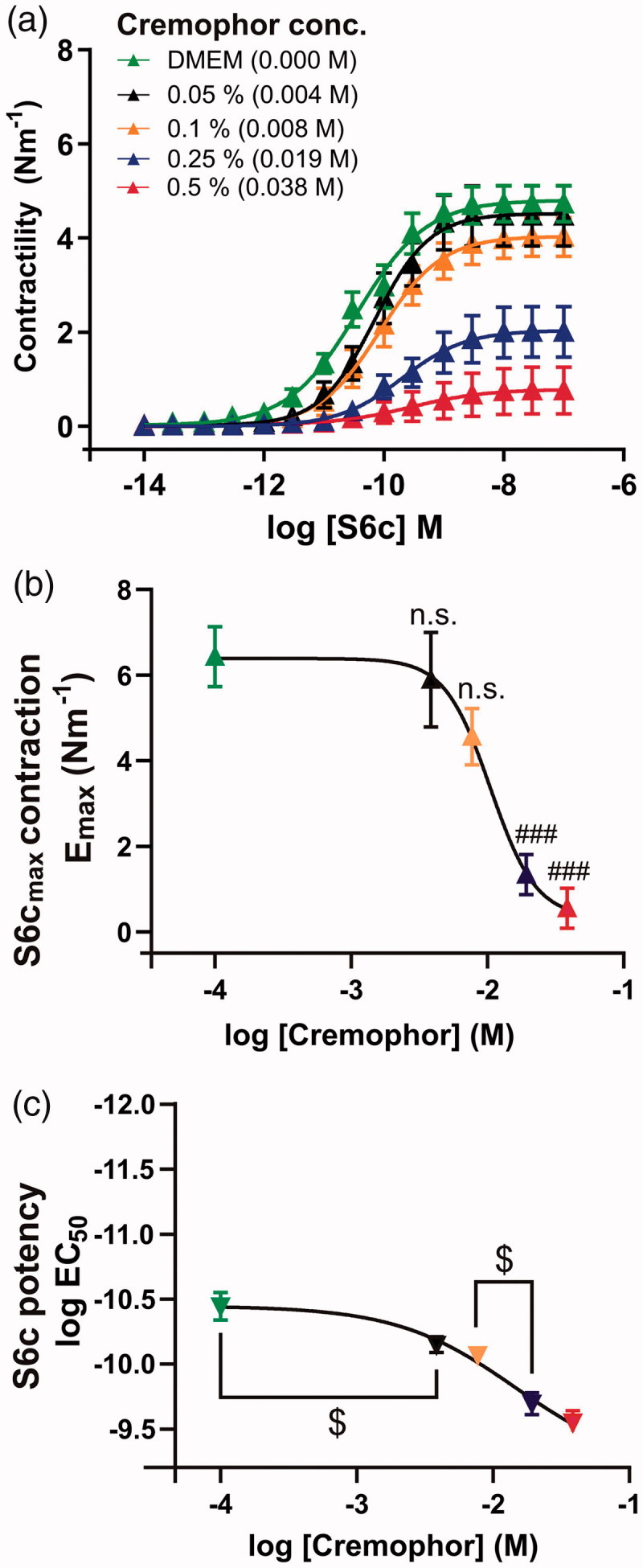
Concentration-dependence of cremophor on S6c-induced contractility after 48 h organ culture. Basilar arteries were incubated with different concentrations of cremophor. (a) Concentration-response curves with S6c show varying log EC_50_ and E_max_ values. Correlation between varying concentrations of cremophor with E_max_ (b) and log EC_50_ (c) from the concentration-response curves with S6c. Values are listed in the Table S2, one-way ANOVA with Holm-Sidak post-test (###*p* < .001). $=non-overlapping 95% confidence intervals. Data are expressed as mean ± SEM, *n* = 6–7.

Since DMSO was long ago shown not to be suitable for drug delivery for human therapies (Smith et al., 1967) and this still remains a concern (Weaver et al., 2016), we examined other clinical studies involving MEK1/2 inhibitors. We found an abundance of available data, in particular from the cancer field, where cremophor often is used as a vehicle and drug delivery system (Gelderblom et al., 2001). Although it was originally thought that cremophor was inert, there are some studies that indicate that it might have minor biological effects. Therefore, we tested four concentrations of cremophor in our organ culture model (Figure 2(a)). All concentrations caused a significant shift in the log EC_50_ value for S6c, compared to the control (Table S2). Concentrations of cremophor 0.25% (0.019 M) or higher had significant effects on the E_max_ (Figure 2(b)). Cremophor inhibition was found to have similar log IC_50_ value for its ability to affect S6c E_max_ (−2.12 to −1.56 log M, Figure 2(b)) and S6c potency (−2.19 to 1.76 log M, Figure 2(c)).

### Effects on general contractility

**Figure 3.**
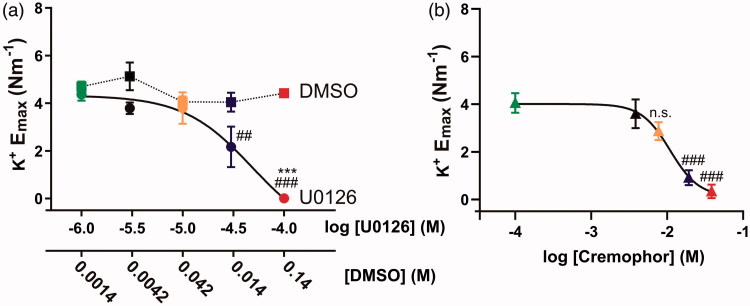
Concentration-dependence of U0126, DMSO, and cremophor on potassium-induced contractility after 48 h organ culture. Basilar arteries were incubated with different concentrations of U0126, corresponding concentrations of DMSO and cremophor. Correlation between the maximal 60 mM potassium contraction (K^+^ E_max_) and the concentrations of U0126/DMSO (a) and cremophor (b). Values are listed in the Table S3, one-way ANOVA with Holm-Sidak post-test (##*p* < .01 and ###*p* < .001) or multiple *t*-tests with Holm-Sidak correction for multiple testing (***p < .001). Data are expressed as mean ± SEM, *n* = 4–15.

**Figure 4.**
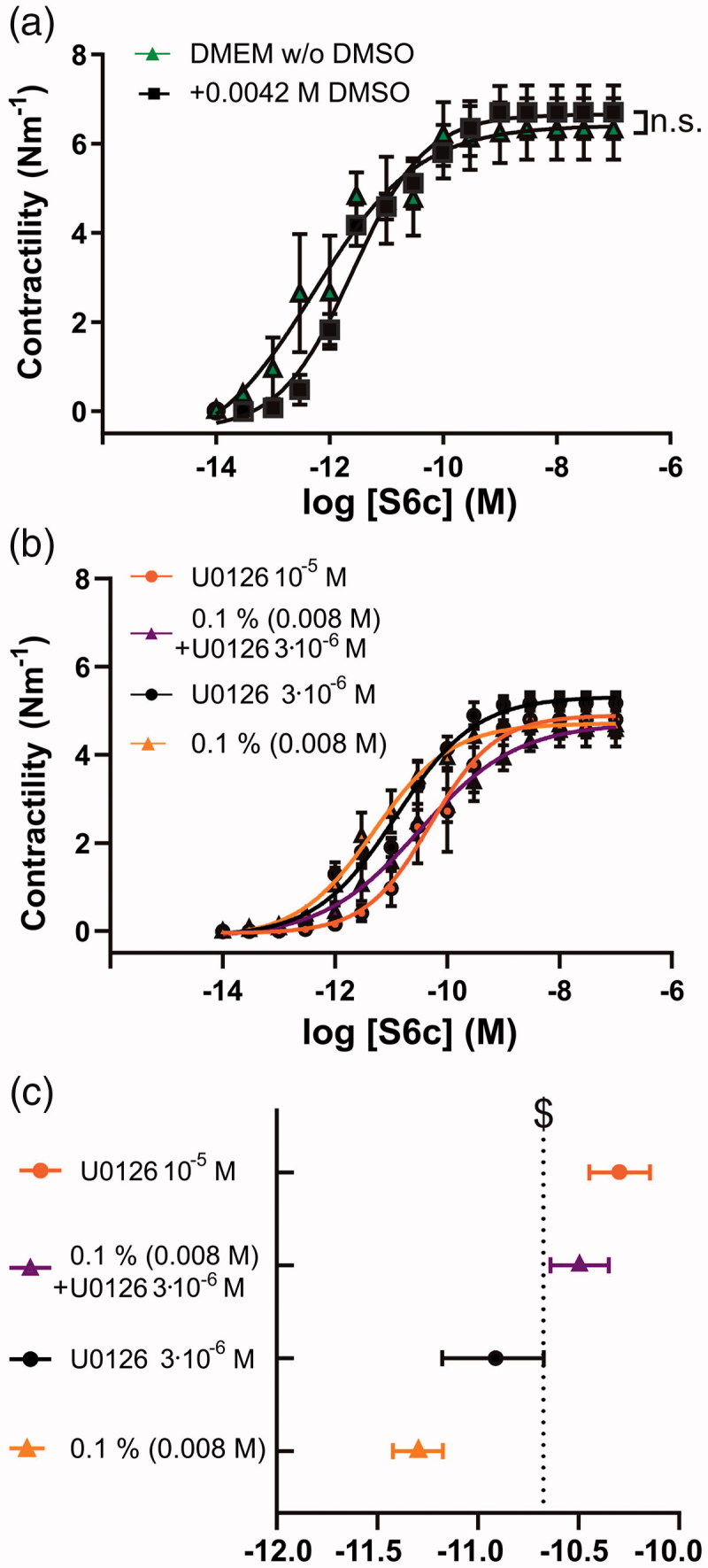
Additive effect of cremophor and U0126 on S6c contractility after 48 h organ culture. (a) Basilar arteries were incubated without DMSO and with 0.0042 M DMSO which is the corresponding concentration for the 3 × 10^−6 ^M U0126. (b) Concentration-response curves with S6c of basilar arteries incubated with cremophor and different concentrations of U0126 in DMSO or cremophor. (c) Comparison of the log EC_50_ values of the different concentrations of U0126 in DMSO or cremophor based on the concentration-response curves with S6c. When 0.1% is stated it refers to 0.1% cremophor. $ = non-overlapping 95% confidence intervals. Data are expressed as mean ± SEM, *n* = 6–7.

To further explore the depolarization-induced contractility, we compared the contractions of the basilar arteries in response to the depolarization with 60 mM potassium buffer. Potassium-induced contractions were significantly reduced at 3 × 10^−5^ and 10^−4 ^M of U0126 (Figure 3(a)) compared to the 10^−6 ^M U0126. Only 10^−4 ^M U0126 was significantly different from its paired DMSO control (*p* < .001). The log IC_50_ value for U0126 shifting the maximal potassium contraction (K^+^ E_max_) was relatively high (−4.97 to −3.66 log [U0126] M). For cremophor, the calculated log IC_50_ value for the effect on the K^+^ E_max_ was −2.15 to −1.77 log M (Figure 4(b)), with significant effects seen from 0.25% (0.019 M) or higher. All values can be found in the Table S3.

### Additive effect of U0126 and cremophor

Since both cremophor and U0126 had inhibitory effects on the arterial contractility we postulated that there might be an additive effect, which should be considered when finding the optimal dose *in vivo* for stroke treatment.

We first compared the DMEM without DMSO to DMEM with 0.0042 M DMSO (Figure 4(a)), which was used as a drug delivery for 3 × 10^−6 ^M U0126 in Figure 1(a). There was no difference (*p* > .05). To further investigate any synergistic effects of cremophor combined with U0126, we compared four selected groups: (I) 0.1% (0.008 M) cremophor, (II) 3 × 10^−6 ^M U0126 in 0.1% (0.008 M) cremophor, (III) 3 × 10^−6 ^M U0126 in DMSO, and (IV) 10^−5 ^M U0126 in DMSO (Figure 4(b)). Figure 4(c) shows that 3 × 10^−6 ^M U0126 in 0.1% (0.008 M) cremophor (log EC_50_, −10.64 to −10.35) was significantly more potent for S6c inhibition than both the matched cremophor control (log EC_50_, −11.42 to −11.17), as well as the matched U0126 concentration in DMSO (log EC_50_, −11.15 to −10.65). Even though it has previously been shown that U0126 prevents upregulation of receptor expression levels using both qPCR, immunohistochemistry (Henriksson et al., 2004) and western blot (Li et al., 2012), such minor changes in EC_50_ values observed in the current study are difficult to detect using these quantitative methods (Ahnstedt et al., 2012). We believe that using the highly specific ET_B_ agonist S6c is sufficient to conclude on functional ET_B_ receptor upregulation and therefore we have not pursued studying ET_B_ receptor expression levels further.

**Figure 5.**
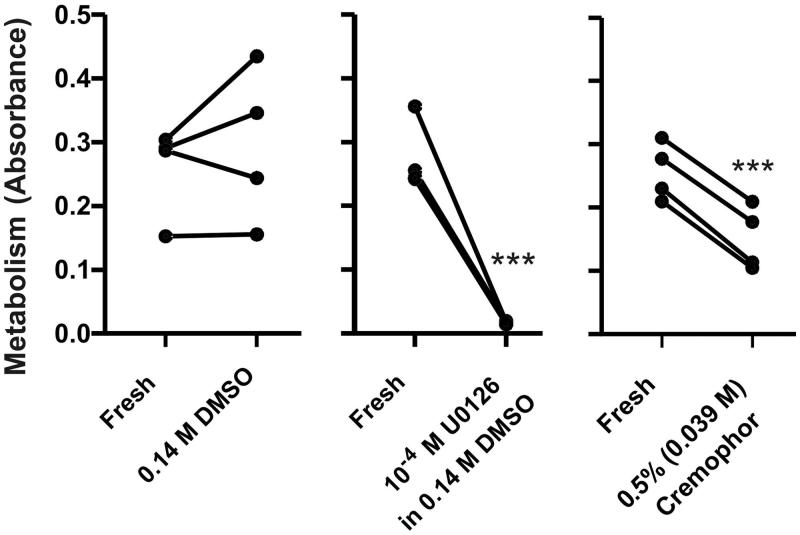
Comparison of metabolism between fresh arteries and after 48 h organ culture. Metabolism was measured using cell counting kit 8. There was no change in the control arteries with DMSO (0.14 M) only. U0126 significantly lowered the metabolism. 0.5% (0.039 M) cremophor also lowered the metabolism but to a less extent than 10^−4 ^M U0126. *n* = 4, paired *t*-test (***p < .001).

In order to evaluate if the lack of contractility seen at the highest concentration of U0126 (100 µM) and cremophor (0.5%, 0.039 M) also affected the cell metabolism, we analyzed BAs at baseline (fresh) and after 48 h in organ culture. There were no significant differences in the metabolism in the fresh BAs from all three groups. Following 48 h there was no change in the metabolism (12.2 ± 12.4%) in the DMSO (0.14 M) control. BAs incubated with 100 µM U0126, had the metabolism reduced by 93.4 ± 0.7% of its internal control. In relation to the cremophor, the metabolism was also reduced, but only with 42.3 ± 4.7% compared to its control. The absolute absorbance values and intra sample trend can be seen in Figure 5.

### *In vivo* toxicology

Based on the obtained data so far, the maximal dose in cerebral spinal fluid (CSF) should not be higher than 3 × 10^−6 ^M U0126 and 0.1% (0.008 M) cremophor. Since the CSF volume in rats is estimated to be around 90 µl (Pardridge, 2011), an intrathecal injection of 15 µl of 1 µM, 10 µM or 20 µM in 0.5% cremophor was chosen to be in coherence with the above parameters for further studies.

We obtained *in vivo* toxicology data on neurological markers, observations (once daily), body weights (prior to the first dose and at necropsy), food consumption (once weekly), and functional observational battery testing (FOB). There were four groups of animals (both male and female subgroups): (I) 0.5% (0.039 M) cremophor alone, (II) low (10^−6 ^M), (III) middle (10^−5 ^M), and (IV) high (2 × 10^−5 ^M) dose of U0126, all in 0.5% (0.039 M) cremophor. Although the value of systemic toxicology study on i.c.v. administration is limited, we still include the table showing that there were no observed differences across treatment groups regarding body weight, food consumption, or organ weights (Table S4).

In the neurological observations the 0.5% cremophor, low and middle dose of U0126 were well tolerated and we observed no changes to any parameters in these groups. At the highest dose of U0126, there were three (two females and one male) out of 16 animals demonstrating clear neurologic signs. Two high-dose females showed signs of neurological impairment during day 6 with FOB testing: one high-dose female was first noted to have a right head tilt and was circling in a clockwise direction before the second daily dose on day 2. This animal had a head tilt for the duration of the study, also noted during day 6 with FOB testing, but the circling movements were only observed in conjunction with the second dose. Another high dose female was noted to have hindquarters rotating clockwise (hind feet circling with forepaws stepping in one spot) during day 6 with FOB testing. Neither animal showed any other abnormal signs during day 6 with FOB testing. One high-dose male showed slight palpebral closure (∼ ¼) during dosing on day 7, but the animal had not shown any abnormality during day 6 with FOB testing. None of the other animals had any abnormal FOB testing results, and the behavioral test results were virtually identical in these animals.

Histological changes, consisting primarily of inflammation of varying severity, were observed in the right lateral ventricle (injection site) and to a lesser extent, the contralateral ventricle of brains from animals in all groups. Small multifocal aseptic abscesses were observed in association with the injection site. Variable multifocal subacute inflammation was also noted at the other brain section levels in the neuropil and in the meninges. The incidence, distribution, and severity of inflammation were similar in all groups (Table 1) and most likely attributed to the procedure of the catheter insertion and not related to the injection. There were no histopathological findings in any other organs.

**Table 1.** Summary of histology inflammation scores.

Groups	Cremophor 0.5%		Low dose U0126		Middle dose U0126		High dose U0126	
Gender	Male	Female	Male	Female	Male	Female	Male	Female
Inflammation score at								
Injection site	2.6 ± 1.0	3.3 ± 0.9	3.5 ± 0.8	2.9 ± 0.8	2.5 ± 0.8	3.0 ± 0.8	3.0 ± 0.9	3.3 ± 0.7
Level 1	0.5 ± 0.9	2.0 ± 0.9	2.1 ± 1.0	1.8 ± 0.7	1.5 ± 0.9	2.0 ± 0.0	2.1 ± 0.4	2.3 ± 1.0
Level 2	1.3 ± 1.0	1.6 ± 1.1	2.6 ± 0.7	2.3 ± 0.5	2.4 ± 0.5	2.3 ± 0.5	3.0 ± 0.8	2.6 ± 0.5
Level 3	1.3 ± 1.0	1.6 ± 1.1	2.4 ± 0.7	2.1 ± 0.4	2.3 ± 0.5	2.0 ± 0.0	2.0 ± 0.0	2.5 ± 0.5
Level 4	1.4 ± 1.2	1.3 ± 1.0	2.3 ± 0.5	2.0 ± 0.0	2.3 ± 0.5	2.0 ± 0.0	2.0 ± 0.0	2.5 ± 0.5
Frequency of histology inflammation scores at brain levels	Cremophor 0.5%		Low dose U0126		Middle dose U0126		High dose U0126	
Injection site								
Normal	0/14		0/16		0/14		0/16	
Score 2	5/14		4/16		6/14		4/16	
Score 3	2/14		5/16		5/14		6/16	
Score 4	7/14		7/16		3/14		6/16	
Abscess	4/14		6/16		2/14		4/16	
Level 1								
Normal	5/14		1/16		2/14		1/16	
Score 2	8/14		12/16		12/14		10/16	
Score 3	1/14		3/16		0/14		5/16	
Score 4	0/14		0/16		0/14		0/16	
Abscess	0/14		0/16		0/14		0/16	
Level 2								
Normal	3/14		0/16		0/14		0/16	
Score 2	10/14		10/16		11/14		6/16	
Score 3	1/14		5/16		3/14		8/16	
Score 4	0/14		1/16		0/14		2/16	
Abscess	0/14		0/16		1/14		0/16	
Level 3								
Normal	3/14		0/16		0/14		3/16	
Score 2	10/14		13/16		13/14		12/16	
Score 3	1/14		2/16		1/14		4/16	
Score 4	0/14		1/16		1/14		0/16	
Abscess	0/14		0/16		0/14		0/16	
Level 4								
Normal	4/14		0/16		0/14		0/16	
Score 2	10/14		14/16		14/14		12/16	
Score 3	1/14		2/16		1/14		4/16	
Score 4	0/14		0/16		0/14		0/16	
Abscess	0/14		0/16		0/14		0/16	

The toxicology study on the i.c.v. administration indicate that 0.5% (0.0039 M) cremophor alone (in aCSF) and 0.5% (0.0039 M) cremophor combined with 10^−5 ^M U0126 are both well tolerated. Compound-related CNS toxicity was observed initially before the second dose on day 2 (i.e. prior to the 4th dose) at the high dose (2 × 10^−5 ^M). Hence the toxicology data support our *ex vivo* organ culture study, suggesting that the maximal i.c.v. administration injected *in vivo* dose should be 0.5% cremophor (in aCSF) combined with 10^−5 ^M U0126, resulting in an effective concentration of around 2 × 10^−6 ^M U0126 in 0.1% cremophor.

## Discussion

In this study, we have investigated the optimal concentration of the MEK1/2 inhibitor U0126 and a suitable *in vivo* vehicle and drug delivery system (cremophor) for inhibiting the functional upregulation of ET_B_ receptors. The optimal dose of U0126 was further tested in a toxicology study. Below we will discuss the possible interaction between U0126 and cremophor, and its relation to possible dose selection for *in vivo* studies.

### U0126

There is strong evidence on the importance of the MEK/ERK1/2 pathway following both *ex vivo* experiments with arterial organ culture and *in vivo* stroke models (Edvinsson & Povlsen, 2011). Despite the use in several papers, also *in vivo*, the optimal dose has not been determined in detail. Thus, we used several different concentrations of U0126 to estimate the optimal concentration that would inhibit functional ET_B_ receptor upregulation without affecting the depolarization-induced contractility of the BA. Our data show that 1 × 10^−5 ^M of U0126, with DMSO as vehicle and drug delivery method, is the highest concentration with a pure effect on preventing functional ET_B_ receptor upregulation without affecting depolarization-induced contractility *ex vivo,* compared to 10^−6 ^M U0126. The highest concentration of U0126 in organ culture (100 µM) did not only reduce absolute contractility, but also negatively affected cellular metabolism (Figure 5).

### Cremophor as vehicle and drug delivery system

Previous studies applying U0126 in stroke treatment has used DMSO as solvent for the weakly polar U0126 (Larsen et al., 2011; Maddahi et al., 2011; Povlsen & Edvinsson, 2015). Cremophor is a synthetic non-ionic surfactant used to stabilize emulsions of nonpolar materials in water. Cremophor is actually no longer considered inert, and it is, for example, able to modify pharmacokinetic properties of other compounds (Gelderblom et al., 2001; Liu et al., 2016) and to specifically inhibit PKC function (Zhao et al., 1989). Even DMSO itself is not inert and has been shown to have positive effects on stroke outcome (Shimizu et al., 1997; Bardutzky et al., 2005). Since DMSO was long ago shown to not be suitable as a vehicle and for drug delivery of human therapies (Smith et al., 1967) and still remains a concern (Weaver et al., 2016), we nevertheless needed a vehicle and drug delivery system for possible human therapy. With the change from DMSO to cremophor in stroke treatments, it is important to determine possible interactions. We chose to study this initially in an *ex vivo* study.

In the literature, there are some studies on the toxicity of cremophor, mainly performed in cell culture. The current dose applied in our study was 5.15 mg/mL (0.5%, 0.039 M) with an effective concentration lower than 0.1% (see below). In epithelial cells, concentrations of 5 mg/mL and above were toxic at incubation times relevant to the present study, while in endothelial cell culture cremophor had negative effects at concentrations higher than 0.1 mg/mL (Kiss et al., 2013). The threshold in the current study seems to be similar, as we observed significant effects on the potassium-induced contractility at concentrations that were at 2.56 mg/mL (0.25%, 0.019 M) or higher (Figure 3(b)). In addition, only a modest decrease in cellular metabolism was observed after 48 h of BA incubation with 0.5% (0.039 M) cremophor, showing that this concentration is not toxic (Figure 5). Compared to the *in vivo* conditions, the injected volume will be diluted in CSF, and therefore we conclude that 0.5% cremophor in the injected solution is the recommended vehicle and drug delivery method, as this is diluted to around 0.1% or lower in the CSF (15 µL into 90 µL (Pardridge, 2011)).

### Synergistic effect

In previous studies investigating possible additive effects of cremophor, it has been shown that cremophor at a concentration as low as 0.01 mg/mL could have enhancing effects when used as a vehicle and drug delivery method. In a study by Tiemann et al., cremophor did not increase the ‘tissue factor pathway inhibitor’ release from endothelial cell cultures alone, but the release was enhanced 2–4 fold after co-stimulation with the calcium ionophore A 23187 (Tiemann et al., 1997).

As mentioned above, cremophor has also been shown to specifically inhibit PKC, as cremophor is an agent that form complexes with DAG (diacylglycerol) and thereby prevents PKC activation (Zhao et al., 1989). This has high importance in relation to phenotypical changes during organ culture, as PKC inhibition has been shown to attenuate ET_B_ receptor upregulation, and it has even been shown to possibly improve stroke outcome (Henriksson et al., 2007).

In the current study, we observed significant effects of cremophor at the lowest concentration applied (0.05%, 0.0515 mg/mL, 3.8 mM), while the inhibitory effect on PKC in a cell assay, was observed at around 1 µM (Zhao et al., 1989). We therefore postulate, that the initial shift in pEC_50_ with the addition of cremophor (Figure 2), compared to DMEM control, might be a minor specific PKC effect. Further inhibition of contractility occurred at the higher concentrations of cremophor, which we speculate is more likely to be due to direct effects of cremophor on the cellular membranes, as it completely prevents contractility but has milder effects on metabolism (Figure 5).

### Toxicology in rodents

Due to the possible strong reduction in contractility *ex vivo* of both cremophor or high concentrations of U0126, we obtained data pertaining to U0126 toxicology of the optimal inhibitory dose in rodents. Three of 16 animals at high dose U0126 exposure demonstrated neurological signs and moderate to severe histological scores, suggesting possible localized toxicity in the brain in these few animals. There were however, animals with moderate to severe histological scores in all groups (both 0.5% cremophor alone or combined with U0126), that did not show any neurological or other clinical signs of toxicity (Table 1), supporting that this is due to the procedure.

The incidence, distribution, and severity of inflammation were similar in all groups, suggesting that the inflammation may be more related to vehicle and drug delivery system tolerability, such as the increase in liquid volume within the ventricular compartment and/or the i.c.v. cannula implantation procedure, rather than direct test article-related neurotoxicity. Of note, similar inflammation changes in the ventricles were also found in beagle dogs, where groups injected with aCSF, 0.5% cremophor, or U0126 + 0.5% cremophor (LE, communication) were all comparable, excluding it from being a cremophor specific effect and supporting it being a procedural effect.

### Clinical relevance

The rationale for the i.c.v treatment is to give the compound as close as possible to the arteries/arterioles in the brain. Generally, the i.c.v. approach is not desirable due to the risks associated with inserting a catheter. Nevertheless, when a patient is diagnosed with SAH, such a catheter is already inserted in the current operation protocols to release intracranial pressure. Hence the injection of the MEK1/2 inhibitor U0126 can be given in the early phases of the SAH.

U0126 is a potent inhibitor of functional ET_B_ receptor upregulation, and we have seen that the concentration with an optimal effect (Figure 1) and with minimal effect on the depolarization-induced contractility (Figure 3) was around 10^−5 ^M U0126 in DMSO. When cremophor was chosen as a vehicle and drug delivery method, it had an additive effect (Figure 4(b)). Hence injection of 10^−5 ^M U0126 in 0.5% cremophor seems to be a dose that will be close to the maximal inhibition *in vivo* without exhibiting side effects, such as effects on the depolarization-induced contractility. This is supported by the toxicology studies, where a dose at 2 × 10^−5 ^M caused minor neurological side-effects in some of the rats.

The current work was used to determine the dose of and vehicle of the U0126 treatment, and lead to the preparations of an *in vivo* study, which was just published (Christensen et al., 2019). This *in vivo* SAH study by Christensen et al. (Christensen et al., 2019), which applied an established rodent model, showed that 10 µM of U0126 + 0.5% cremophor improves the outcome following SAH over the current nimodipine treatment. Furthermore, we indeed did observe a positive effect of the cremophor drug delivery system *in vivo*, confirming the relevance of the current study.

## Conclusion

In conclusion, concentrations up to 10^−5 ^M U0126 in 0.5% cremophor and 0.5% cremophor alone are well tolerated at doses that have an effect on functional ET_B_ receptor upregulation in an organ culture model. In addition, cremophor has a synergistic effect preventing functional upregulation of ET_B_ receptors, but not any known additional side effects which make it a suitable vehicle and drug delivery system. Further, both U0126 and cremophor are well tolerated *in vivo* (the toxicology test) at doses showing effects in the *ex vivo* experiments. These data will be important in determining the optimal dose for humans in treating SAH.

## Supplementary Material

Supplemental Material
