# Supplementary material for: Synergistic effects of a cremophor EL drug delivery system and its U0126 cargo in an ex vivo model
Source: Drug Deliv. 2019 Jul 5;26(1):680–8. doi: 10.1080/10717544.2019.1636421 (PMC6691891; doi:10.1080/10717544.2019.1636421)
Supplement: Supplemental Material [file IDRD_A_1636421_SM9686.zip › S1_Table.docx]

**S1 Table. Data on S6c E_MAX_ and EC_50_ for U0126 and DMSO**

| **U0126 (M)** | **DMSO (M)** | **U0126 S6c E_MAX_** | **DMSO S6c E_MAX_** | **Adjusted P-value** | | | **n** |  |  |
| --- | --- | --- | --- | --- | --- | --- | --- | --- | --- |
| 10^-6^ | 0.0014 | 5.41 ± 0.44 | 5.61 ± 0.21 | 0.89 | | | 15/12 |  |  |
| 3·10^-6^ | 0.0042 | 5.17 ± 0.17 | 6.71 ± 0.60 | 0.183 | | | 4 |  |  |
| 10^-5^ | 0.014 | 5.80 ± 0.62 | 5.07 ± 0.42 | 0.89 | | | 4 |  |  |
| 3·10^-5^ | 0.042 | 2.57 ± 1.08 | 5.61 ± 0.78 | 0.183 | | | 4 |  |  |
| 10^-4^ | 0.14 | 0.05 ± 0.04 | 5.45 ± 0.26 | < 0.001* | | | 4 |  |  |
|  |  |  |  |  | |  | |  |  |
| **U0126 (M)** | **DMSO (M)** | **U0126 S6c EC_50_ ^¤^** | **DMSO S6c EC_50_ ^¤^** | **n** |  |  |  |  |  |
| 10^-6^ | 0.0014 | -11.57 to -10.72 | -11.31 to -10.83 | 15/12 |  |  |  |  |  |
| 3·10^-6^ | 0.0042 | -11.15 to -10.65 | -11.83 to - 11.35* | 4 |  |  |  |  |  |
| 10^-5^ | 0.014 | -10.45 to -10.15 | -11.61 to -11.32* | 4 |  |  |  |  |  |
| 3·10^-5^ | 0.042 | -10.14 to -10.05 | -12.09 to -11.64* | 4 |  |  |  |  |  |
| 10^-4^ | 0.14 | N.D. | -11.70 to -11.31 | 4 |  | |  |  |  |

***Significant compared to paired DMSO control,** ^¤^**Data for log EC_50_ are the range of the 95 % confidence intervals.**
