# Supplementary material for: Synergistic effects of a cremophor EL drug delivery system and its U0126 cargo in an ex vivo model
Source: Drug Deliv. 2019 Jul 5;26(1):680–8. doi: 10.1080/10717544.2019.1636421 (PMC6691891; doi:10.1080/10717544.2019.1636421)
Supplement: Supplemental Material [file IDRD_A_1636421_SM9686.zip › S2_Table.docx]

**S2 Table. Data on S6c E_MAX_ and EC_50_ for Cremophor**

| **Cremophor (M)** | **Cremophor (%)** | **S6c E_MAX_** | **Adjusted P-value** | **n** |  |
| --- | --- | --- | --- | --- | --- |
| 0 | 0 | 6.43 ± 0.70 | - | 7 |  |
| 0.004 | 0.05 | 5.90 ± 1.11 | 0.5972 | 6 |  |
| 0.008 | 0.1 | 4.56 ± 0.66 | 0.1381 | 6 |  |
| 0.019 | 0.25 | 1.34± 0.47 | <0.001^#^ | 6 |  |
| 0.039 | 0.5 | 0.55 ± 0.46 | <0.001^#^ | 7 |  |
|  |  |  |  |  |  |
| **Cremophor (M)** | **Cremophor (%)** | **S6c EC_50_^¤^** | **Significant vs. 0 M** | **n** |  |
| 0 | 0 | -10.55 to -10.34 | - | 7 |  |
| 0.004 | 0.05 | -10.21 to -10.09^#^ | Yes | 6 |  |
| 0.008 | 0.1 | -10.10 to -10.03^#^ | Yes | 6 |  |
| 0.019 | 0.25 | -9.78 to -9.61^#^ | Yes | 6 |  |
| 0.039 | 0.5 | -9.64 to -9.46^#^ | Yes | 7 |  |

**^#^Significant compared to control (0M cremophor),** ^¤^**Data for log EC_50_ are the range of the 95 % confidence intervals.**
