# Supplementary material for: Synergistic effects of a cremophor EL drug delivery system and its U0126 cargo in an ex vivo model
Source: Drug Deliv. 2019 Jul 5;26(1):680–8. doi: 10.1080/10717544.2019.1636421 (PMC6691891; doi:10.1080/10717544.2019.1636421)
Supplement: Supplemental Material [file IDRD_A_1636421_SM9686.zip › S3_Table.docx]

**S3 Table. Data on K^+^ E_MAX_ and EC_50_ for U0126 and DMSO**

| **U0126 (M)** | **DMSO (M)** | | **U0126 K^+^ E_MAX_** | | **DMSO K^+^ E_MAX_** | | **Adjusted P-value** | | **n** | |  |  |  |  |  |
| --- | --- | --- | --- | --- | --- | --- | --- | --- | --- | --- | --- | --- | --- | --- | --- |
| 10^-6^ | 0.0014 | | 4.37 ± 0.26 | | 4.69 ± 0.21 | | 0.55 | | 15/12 | |  |  |  |  |  |
| 3·10^-6^ | 0.0042 | | 3.79 ± 0.24 | | 5.13 ± 0.58 | | 0.27 | | 4 | |  |  |  |  |  |
| 10^-5^ | 0.014 | | 3.79 ± 0.66 | | 4.06 ± 0.23 | | 0.72 | | 4 | |  |  |  |  |  |
| 3·10^-5^ | 0.042 | | 2.17 ± 0.85 | | 4.05 ± 0.40 | | 0.27 | | 4 | |  |  |  |  |  |
| 10^-4^ | 0.14 | | 0.01 ± 0.01 | | 4.42 ± 0.04 | | <0.001* | | 4 | |  |  |  |  |  |
|  | |  | |  | |  | |  | | | |  | |  |  |
| **Cremophor (M)** | | **Cremophor (%)** | | **K^+^ E_MAX_** | | **Adjusted P-value** | | **n** | |  | | |  |  |  |
| 0 | | 0 | | 4.05 ± 0.41 | | - | | 7 | |  | | |  |  |  |
| 0.004 | | 0.05 | | 3.60 ± 1.60 | | 0.44 | | 6 | |  | | |  |  |  |
| 0.008 | | 0.1 | | 2.87 ± 0.38 | | 0.096 | | 6 | |  | | |  |  |  |
| 0.019 | | 0.25 | | 0.91± 0.32 | | <0.001^#^ | | 6 | |  | | |  |  |  |
| 0.039 | | 0.5 | | 0.34 ± 0.28 | | <0.001^#^ | | 7 | |  | | |  |  |  |

***Significant compared to paired DMSO control, ^#^Significant compared to control (0 M cremophor).**
