# Supplementary material for: Synergistic effects of a cremophor EL drug delivery system and its U0126 cargo in an ex vivo model
Source: Drug Deliv. 2019 Jul 5;26(1):680–8. doi: 10.1080/10717544.2019.1636421 (PMC6691891; doi:10.1080/10717544.2019.1636421)
Supplement: Supplemental Material [file IDRD_A_1636421_SM9686.zip › S4_Table.docx]

**S4 Table. Body weights, organ weights and food consumption**

| **Group** | **Cremophor 0.5 %** | | **Low dose U0126** | | **Middle dose U0126** | | **High dose U0126** | |
| --- | --- | --- | --- | --- | --- | --- | --- | --- |
| **Gender** | **Male** | **Female** | **Male** | **Female** | **Male** | **Female** | **Male** | **Female** |
| **Body weights (g), Day 0** | 248.8 ± 17.3 | 233.3 ± 13.1 | 248.6 ± 14.7 | 232.2 ± 11.5 | 252.5 ± 15.5 | 229.4 ± 11.6 | 247.1 ± 15.5 | 232.6 ± 10.4 |
| **Body weights (g), Day 7** | 263.9 ± 22.2 | 238.8 ± 15.2 | 263.9 ± 23.9 | 235.1 ± 10.1 | 269.6 ± 11.9 | 235.8 ± 11.3 | 251.7 ± 23.0 | 233.0 ± 9.2 |
| **Body weights Normalized (% Day 0), Day 7** | 106.8 ± 5.3 | 102.3 ± 4.5 | 106.0 ± 4.8 | 101.3 ± 3.5 | 105.3 ± 2.7 | 102.9 ± 4.3 | 102.0 ± 9.4 | 100.2 ± 3.3 |
| **Carcass weights (g)** | 264.5 ± 20.6 | 238.8 ± 15.2 | 263.9 ± 23.9 | 235.1 ± 10.1 | 269.6 ± 11.9 | 235.8 ± 11.3 | 251.7 ± 23.0 | 233.0 ± 9.2 |
| **Adrenals (pr) weights (g)** | 0.08 ± 0.02 | 0.11 ± 0.02 | 0.08 ± 0.04 | 0.11 ± 0.02 | 0.07 ± 0.02 | 0.12 ± 0.03 | 0.09 ± 0.02 | 0.11 ± 0.02 |
| **Brain weights (g)** | 1.83 ± 0.12 | 1.87 ± 0.06 | 1.92 ± 0.10 | 1.81 ± 0.17 | 1.96 ± 0.10 | 1.91 ± 0.09 | 1.94 ± 0.08 | 1.90 ± 0.08 |
| **Heart weights (g)** | 1.02 ± 0.17 | 0.96 ± 0.11 | 1.08 ± 0.14 | 0.97 ± 0.06 | 1.02 ± 0.09 | 0.99 ± 0.09 | 1.10 ± 0.07 | 0.96 ± 0.07 |
| **Kidneys (pr) weights (g)** | 2.39 ± 0.34 | 2.20 ± 0.20 | 2.52 ± 0.39 | 2.14 ± 0.11 | 2.47 ± 0.21 | 2.23 ± 0.21 | 2.35 ± 0.17 | 2.09 ± 0.20 |
| **Liver weights (g)** | 13.06 ± 1.89 | 11.50 ± 0.86 | 13.74 ± 2.01 | 10.72 ± 0.65 | 14.05 ± 0.91 | 10.94 ± 0.76 | 12.29 ± 1.78 | 11.23 ± 0.85 |
| **Pituitary weights (g)** | 0.0052 ± 0.0021 | 0.0055 ± 0.0038 | 0.0045 ± 0.0034 | 0.0076 ± 0.0037 | 0.008 ± 0.0041 | 0.0083 ± 0.0062 | 0.0046 ± 0.0029 | 0.0058 ± 0.0024 |
| **Prostate weights (g)** | 0.20 ± 0.08 |  | 0.15 ± 0.07 |  | 0.23 ± 0.04 |  | 0.16 ± 0.03 |  |
| **Testes (pr) weights (g)** | 3.16 ± 0.28 |  | 3.18 ± 0.18 |  | 3.44 ± 0.09 |  | 3.15 ± 0.14 |  |
| **Ovaries (pr) weights (g)** |  | 0.15 ± 0.02 |  | 0.14 ± 0.05 |  | 0.14 ± 0.03 |  | 0.13 ± 0.02 |
| **Spleen weights (g)** | 0.69 ± 0.03 | 0.69 ± 0.09 | 0.69 ± 0.11 | 0.65 ± 0.09 | 0.69 ± 0.07 | 0.69 ± 0.10 | 0.70 ± 0.10 | 0.70 ± 0.06 |
| **Thyroids & Parathyroids (pr) weights (g)** | 0.017 ± 0.007 | 0.021 ± 0.004 | 0.021 ± 0.008 | 0.015 ± 0.007 | 0.023 ± 0.004 | 0.022 ± 0.012 | 0.019 ± 0.012 | 0.017 ± 0.009 |
| **Food consumption (7 days)** | | | | | | | | |
| **Net food consumption (g)** | 154.1 ± 26.8 | 152.3 ± 29.5 | 161.8 ± 34.5 | 129.7 ± 10.1 | 151.4 ± 62.5 | 146.2 ± 22.0 | 138.2 ± 22.0 | 146.8 ± 32,2 |
